# Differential Impact of Nitric Oxide and Abscisic Acid on the Cellular and Physiological Functioning of *sub1A* QTL Bearing Rice Genotype under Salt Stress

**DOI:** 10.3390/plants11081084

**Published:** 2022-04-15

**Authors:** Indraneel Saha, Arijit Ghosh, Debabrata Dolui, Masayuki Fujita, Mirza Hasanuzzaman, Malay Kumar Adak

**Affiliations:** 1 Plant Physiology and Plant Molecular Biology Research Unit, Department of Botany, University of Kalyani, Kalyani 74 1235, India; indraneelsaha92@gmail.com (I.S.); arijitgo625@gmail.com (A.G.); debabratabotany@gmail.com (D.D.); 2 Laboratory of Plant Stress Responses, Department of Applied Biological Science, Faculty of Agriculture, Kagawa University, 2393 Ikenobe, Miki-cho, Kita-gun, Kagawa 761-0795, Japan; 3 Department of Agronomy, Faculty of Agriculture, Sher-e-Bangla Agricultural University, Sher-e-Bangla Nagar, Dhaka 1207, Bangladesh

**Keywords:** abiotic stress, antioxidant defense, osmotic potential, oxidative stress, phytohormone signaling, neurotransmitters, reactive oxygen species, polyamines

## Abstract

Hydroponic culture containing 200 mM NaCl was used to induce oxidative stress in seedlings of cultivars initially primed with 1 mM SNP and 10 µM ABA. Exogenous application of sodium nitroprusside (SNP – a nitric oxide donor) and abscisic acid (ABA) was well sensitized more in cv. Swarna Sub1 than cv. Swarna and also reflected in different cellular responses. The major effects of salinity, irrespective of the cultivar, were lowering the water relation, including relative water content and osmotic potential, and decreasing the compatible solutes like alanine, gamma-aminobutyric acid, and glycine betaine. The accumulated polyamines were reduced more in cv. Swarna with a concomitant decrease in photosynthetic reserves. NADP-malic enzyme activity, sucrose accumulation, ascorbate peroxidase, and glutathione *S*-transferase activities gradually declined under NaCl stress and the catabolizing enzymes like invertase (both wall and cytosolic forms) also declined. On the contrary, plants suffered from oxidative stress through superoxide, hydrogen peroxide, and their biosynthetic enzymes like NADP(H) oxidase. Moderation of Na^+^/K^+^ by both SNP and ABA were correlated with other salt sensitivities in the plants. The maximum effects of SNP and ABA were found in the recovery of antioxidation pathways, osmotic tolerance, and carbohydrate metabolism. Findings predict the efficacy of SNP and ABA either independently or cumulatively in overcoming NaCl toxicity in rice.

## 1. Introduction

Chemical elicitation has been one of the feasible measures for crop plants to diminish or mitigate abiotic stress factors. A number of chemical residues acting as elicitors, which are mostly analogous to plants’ metabolites, have been used to moderate the stress perception (its signaling, induction of selected gene(s), and finally, the manifestation of physiological responses). For each elicitor residue, significant variations can be observed among the stress tolerant and sensitive cultivars through downstream cellular and physiological responses. Those elicitors are in-built residues in plant cells actively engaged in existing metabolic pathways and often identified as effective in many crop species. Nitric oxide (NO) is one that is principally a derivative of reactive nitrogen species (RNS) and is produced as a byproduct of alteration of nitrate and nitrite reduction. It is a highly diffusible and multipurpose inter/intra-signaling residue involved in osmotic balance, membrane transport, and antioxidation under abiotic stresses such as water deficits [1], salinity [2], hypoxia/anoxia [3], and metal and xenobiotic toxicity [4]. A number of endogenous growth regulators, including gibberellic acid (GA), abscisic acid (ABA), ethylene (ET), salicylic acid (SA), polyamines (PAs), jasmonic acid (JA), signaling moieties like Ca^2+^ and cyclic nucleotides, and redox stabilizing glutathione are complementary with NO activity for stress tolerance. Nitric oxide, with its instability in nature and additional free electrons, can easily neutralize the excess intracellular reactive oxygen species (ROS) through reduction with NADP(H) [5]. This is more prevalent in water or salinity stress where oxidative redox may be directly encountered by NO. Thereby, downstream physiological activities like membrane peroxidation, membrane transport, metabolite turnover, and enzyme-substrate saturation are rescued. NO and ABA signaling pathways can often sense ROS metabolism, such as induction and distribution of hydrogen peroxide (H_2_O_2_), following downstream cellular responses facing salt stress [6].

Interestingly, plants that accumulate salts over a threshold cellular concentration also develop RNS. This causes induction of NO accumulation through an altered pathway of nitrite reduction. Alternatively, NO may originate from the PA biosynthetic pool where arginine decarboxylase (ADC) becomes crucial in an autocatalytic pathway with oxygen (O_2_) and NADP(H) [7]. In other ways, excluding a few algal species, there is still dispute on the existence of any principal oxidative NO synthesizing enzyme/synthase (NOE/S) in crop species [8]. For the non-enzymatic path of NO synthesis, a few less frequent residues like *S*-nitrosoglutathione (GSNO) and hyponitrous acid (H_2_N_2_O_2_) deliver NO under water-deficit, as reported in some crops [9]. Furthermore, both NO and ROS (like H_2_O_2_) residues are thought to complement subsequent changes in redox homeostasis as plants progress through water and salinity stress. In some cases, use of sodium nitroprusside (SNP) in high doses has been shown to induce cyanide toxicity in tissues [10]. For those cases, a systematic and local response equivalent to cyanide sensitivity becomes a bottleneck for realizing satisfactory effects from SNP.

On the other hand, the implication of NO in stress recovery is also mediated through induction of other growth regulators where ABA is frequently observed, and even more so under osmotic and salinity stress [11]. In these cases, dehydration-induced NO signaling often matches the rise in ABA and its related biosynthetic and metabolic genes like *9-cis-epoxycarotenoid dioxygenase* (*NCED*) [12]. This leads to an increase in stromal pH of the illuminated chloroplast and release of ABA when plants are primed with NO. A cytosolic increase in Ca^2+^ flux with a low H^+^ gradient over the chloroplast membrane is another impact of NO-governed stomatal regulation of water loss [13]. In more detail, *S*-nitrosylation is the key to initiating stomatal regulation with ABA over accumulation triggered by respiratory burst oxidase homolog (Rboh)-dependent ROS and NO generation by nitrite reductase activity. Therefore, this advocates for ROS and NO activity either in complementary or supplementary mode to maximize the turgor pressure of guard cells through ABA signaling. Within a few years, exogenous application of NO showed significant ameliorating roles in crop species [14]. However, NO application is based on the mitigation of oxidative damage in crops where ABA signaling pathways are predominant. In a combinational study with ABA, a NO donor, giving a direct release of NO, and a NO scavenger were used to validate ABA-induced antioxidation paths covering antioxidative enzymes in forage legumes [15]. NO and ABA interactions also become pertinent in cellular antioxidation paths where reduced glutathione (GSH) binds with NO, forming GSNO. This is hydrolyzed by GSNO reductase to release NO, which restarts the cycle, leading to overexpression of the antioxidative cascade by superoxide dismutase (SOD) and glutathione reductase (GR) activity. ABA biosynthesis was realized in a parallel manner with overexpression of the *NCED* gene in guard cells with exogenous H_2_O_2_ and NO application on maize leaves [16]. Therefore, because of the importance of NO and ABA in signaling following downstream reactions, it is worth considering how these two could be sensed in the wider network of plant adaption to stress conditions.

Rice is especially vulnerable to salinity and impeded growth of plant genotypes has been shown, causing a bottleneck in satisfactory yield. Under salinity, rice is affected primarily by dehydration/water deficit stress and secondarily by specific ion effects with Na^+^ accumulation [17]. Expectedly for the former, rice plants may relieve the water deficits by osmoregulation, where ABA involvement in the hydro-active path is the prerequisite. Therefore, from the above-mentioned elucidation, it is anticipated that NO-mediated ABA function may occur under salinity stress. With this view, we used two rice cultivars, one with special traits with *sub1A* quantitative trait locus (QTL) (cv. Swarna Sub1) and another non *sub1A* bearing (cv. Swarna) one. In lowland rice ecosystems, submergence and drought appear simultaneously in a single crop cycle. However, *sub1A*, an ethylene-responsive factor (ERF), in a few rice accessions, can reduce biosynthesis as well as gibberellic acid responsiveness under water regime quiescence strategies [18]. These aim for a regulated or economic utilization of carbohydrate reserves to limit aerobic energy-yielding metabolism following suppression of plant growth. *sub1A*, an ERF transcription factor found in limited rice accessions, dampens ethylene production and gibberellic acid responsiveness during submergence, economizing carbohydrate reserves and significantly prolonging endurance. *sub1A* QTL possessing factor, which is also amenable to tolerance of dehydration stress, is expressed in a few rice genotypes under submergence and even de-submergence periods [19]. Furthermore, *sub1A*, which is an ERF, comprehends its effect under salinity by sharing a common signaling path with ABA. However, there are very few reports on whether *sub1A* QTL in rice might also be involved with NO independently or dependently under salinity or dehydration stress. Herein, we describe the impact of SNP (a NO donor) and ABA on the independent application or pretreatment for 24 h duration followed by 200 mM sodium chloride (NaCl)-mediated salinity stress for 72 h on 7-day-old seedlings of a *sub1A* possessing rice cultivar (cv. Swarna Sub1) and a non *sub1A* rice cultivar (cv. Swarna). This study elucidates additional insights on the underlying mechanism of the effect of NO and ABA on stress metabolites, spatial ROS distribution, antioxidants, polymorphism in antioxidative expression, organic acid metabolism, etc., conferring the salinity responses.

## 2. Materials and Methods

### 2.1. Establishment of Seedlings and Treatments

Healthy and viable seeds of rice (*Oryza sativa* L.) genotypes viz. cv. Swarna (submergence sensitive) and Swarna Sub1 (submergence tolerant) were germinated following the standard methodology. Initially, seeds were surface sterilized by dipping in 0.01% (*w*/*v*) mercuric chloride solution with gentle agitation for a brief period followed by repeated washing under tap water. Seeds were transferred to sterile deionized water for 5 days until they sprouted under the controlled condition of 37 °C, 80% relative humidity. Following sprouting, 7–10-day-old seedlings of each cultivar were divided into two groups, of which one group was primed with 1 mM of SNP as [SNP (+)] and 10 μM of ABA as [ABA (+)] in a solution of ¼ strength of liquid nutrient media [20] for 24 h separately [21]. Another group (without priming of SNP or ABA) was considered as the control [SNP/ABA (−)]. Thereafter, one set of each group of seedlings was subjected to 200 mM NaCl stress [SS (+)] in the same media for 72 h along with nutrient medium and another set remained without any salinity stress and was considered as [SS (−)]. The experiment was conducted with three replicates per treatment. Finally, all seedlings of [SS (+)] and [SS (−)] were rescued, washed, sampled, frozen in liquid nitrogen, and transferred to cold storage (−80 °C) for further biochemical assays.

### 2.2. Quantification of NO, ABA, and Total PAs

For the determination of NO, plant samples were homogenized in 50 mM acetic acid (pH 3.6), followed by centrifugation at 10,000× *g* at 4 °C for 15 min. Thereafter, the supernatant was decolorized with charcoal, mixed with Griess reagent and the absorbance was taken at 540 nm as suggested by Nahar et al. [22]. ABA quantification was done by high performance liquid chromatography (HPLC). At first, fresh samples were homogenized in 80% methanol and centrifuged at 5000× *g* at 4 °C for 30 min. The supernatant was filtered through a 200 μM membrane filter and injected into the HPLC column (4.6 × 250 mm, C18) coupled with reverse phase using 60% acetonitrile with 0.1% acetic acid as solvent. The flow rate was maintained at 10 μL min^−1^ and the retention time of ABA was standardized at about 5.3 min. Finally, ABA content in the samples was determined from the peak areas against the ABA standard [23]. Separation and quantification of total PA content was done by thin layer chromatography (TLC) as suggested by Young and Galston [24]. Samples were extracted in 5% (*v*/*v*) perchloric acid (HClO_4_) and centrifuged at 12,000× *g* for 15 min at 4 °C. Thereafter, the extracts were separated on a TLC plate (Whatman LK6D) in a solvent system of cyclohexane/ethylacetate (5:4, *v*/*v*) along with standards of PAs. For quantification of PAs, bands were scraped into ethylacetate and the fluorescence was read at 500 nm (excitation) and 350 nm (emission) with the help of a spectrofluorometer.

### 2.3. Determination of Water Status and Metallic Ions

Determination of relative water content (RWC) was done on the basis of fresh, dry and turgid weight of the leaves [25]. Determination of osmotic potential was done based on the gravimetric method and calculated according to Khalid et al. [26]. The determination of Na^+^ and K^+^ was done on the basis of tri-acid mixture (HNO_3_:H_2_SO_4_:HClO_4_, 3:3:1) digestion as suggested by Buschmann et al. [27].

### 2.4. Determination of Alanine, Gamma-Aminobutyric Acid and Glycine Betaine

Determination of compatible solutes such as alanine (ALA), gamma-aminobutyric acid (GABA), and glycine betaine (GB) was done according to the following methods with slight modification. For determination of alanine content, the plant extract was separated with a silica gel chromatogram (Mark) on solvent with butanol: acetic acid: water (1:1:1) against standards with alanine. The eluted amino acid was measured with 1% acid ninhydrin solution reagent reading absorbance at 570 nm [28]. For GABA, the plant sample was extracted in 10% aqueous extract and the soup was centrifuged at 10,000 rpm, 4 °C, 15 min. The soup was concentrated with di ethyl ether, allowed to evaporate and reduction of NADP to NADPH at 340 nm was read and determined by using the content with 1-amnio butyric acid as standard [29]. For glycine betaine content, lyophilized samples were acidified with 2N sulfuric acid, followed by heating at 60 °C overnight. The supernatant was collected after centrifugation, and diluted with acid. The collected pool was reacted with KI-I_2_ solution to precipitate crystal, dissolved with 1,2 di chloroethane, and absorbance was read at 600 nm against standard pure betaine salt [30].

### 2.5. Determination of Carbohydrate Status and Their Polymorphisms

For the assay of NADP-ME, fresh leaf samples were homogenized into a 3 mL extraction buffer consisting of 100 mM Tris-hydrochloric acid (Tris-HCl) (pH 7.3), 10 mM magnesium chloride (MgCl_2_), 10 mM ethylenediaminetetraacetic acid (EDTA), 2 mM dipotassium phosphate (K_2_HPO_4_), 1 mM phenylmethylsulfonyl fluoride (PMSF) and 1 mM β-mercaptoethanol (β-ME) following centrifugation at 12,000× *g*, 15 min, 4 °C. The protein from the extract was partially purified by 80% ammonium sulfate precipitation at 4 °C. Then, 0.1 mL of protein extract was added to 1 mL of reaction buffer containing 60 mM Tris-HCl (pH 8.0), 10 mM EDTA, 10 mM MgCl_2_ and 1 mM NADP-sodium salt. Finally, the content was determined by reading the absorbance at 340 nm as suggested by Ghannoum et al. [31]. The in-gel analysis through non SDS-PAGE (polyacrylamide gel electrophoresis) and its densitometric scanning of the enzyme were done according to Takeuchi et al. [32]. Plant tissue was extracted with 80% (*v*/*v*) ethanol under boiling, and concentrated with a rotary evaporator (Rotavapor R-300, Buchi). The solution was alkaline with KOH and adjusted to pH 5.5 by Na-acetate buffer. From the aliquot, sucrose content was determined according to Anur et al. [33]. For cytosolic and wall-bound invertase, plant samples were extracted with extraction buffer containing 100 mM Tris-HCl (pH 4.8), 10 mM MgCl_2_, 0.1% bovine serum albumin (BSA), 0.1 mM dithiothreitol (DTT) and 0.1 mM PMSF following centrifugation at 12,000× *g* at 4 °C for 15 min. The pellet was re-extracted with the same extraction buffer to 5 mM NaCl. Finally, the enzyme extracts were incubated with 200 mM sucrose and 20 mM sodium citrate buffer (pH 3.8) for 30 min at room temperature, and the activities of both soluble as well as wall-bound invertase (EC 3.2.1.26) were determined by reading the absorbance at 510 nm [34]. The in-gel and its densitometric analysis of invertase activity (wall-bound and soluble) were done as suggested by Ranwala and Miller [35].

### 2.6. Assay of NADP(H) Oxidase and Its Polymorphism

To determine the NADP(H) oxidase (NOX) activity (EC 1.6.3.1), plant samples were homogenized with 50 mM potassium phosphate buffer (pH 6.5) containing 1% (*w*/*v*) polyvinylpyrrolidone (PVP), 0.2 mM EDTA, 5 mM ascorbate, 0.1% (*v*/*v*) triton, 0.1% (*w*/*v*) DTT, 1% (*w*/*v*) leupeptine, 0.1% (*w*/*v*) BSA and subsequently, centrifuged at 10,000× *g*, 4 °C, 20 min, and the supernatant was then extracted with 80% (*w*/*v*) ammonium sulfate precipitation following purification through 65 mM phosphate buffer (pH 7.8), 0.1% (*w*/*v*) BSA, and 0.1% (*w*/*v*) sodium dodecyl sulfate (SDS). An aliquot of enzyme extract was then added in an assay mixture of sodium acetate buffer (pH 7.5), 0.1 mM DTT, 0.1 mM *p*-coumaric acid, 0.05 mM NADP(H), and 5 mM manganese chloride (MnCl_2_), and then the absorbance was read at 340 nm [36]. For the polymorphism through in-gel analysis, the extracted enzyme was run on a 10% non SDS-PAGE at 10 mV/lane in 10 mM Tris-HCl buffer (pH 6.8), followed by washing the gel in phosphate buffer (pH 4.6) and incubating it in the dark at room temperature for 1 h. The solution consisted of 50 mM Tris-HCl, (pH 7.4), 0.2 mM nitroblue tetrazolium (NBT), 0.1 mM MgCl_2_, 1 mM calcium chloride (CaCl_2_) and 0.2 mM NADPH. After that, photographs of the gel were taken in Gel Doc instrument with high resolution following densitometric analysis (Gel Analyzer software (Version-19.1)) [37].

### 2.7. Histochemical Detection and Content of ROS Generation

Analysis of O_2_**^•^**^−^ and H_2_O_2_ generation both in vivo and in vitro was done according to Dunand et al. [38]. Fresh leaves and roots samples were incubated overnight with 50 mM phosphate buffer containing 6 mM NBT salt (pH 4.8) and 5 mM 3,3′-diaminobenzidine (DAB) solution, respectively, for the histochemical detection of O_2_**^•^**^−^ and H_2_O_2_. Thereafter, the distribution of dark blue patches (for O_2_**^•^**^−^) and brown spots (for H_2_O_2_) on leaves and roots were captured by a digital camera (De-winter, India). For determination of O_2_**^•^**^−^ generation, plant samples were extracted with 65 mM phosphate buffer (pH 6.8), followed by centrifugation at 8000× *g* for 30 min at 4 °C. An aliquot of the supernatant with 65 mM phosphate buffer (pH 6.8) and 10 mM hydroxylamine was incubated at room temperature for 45 min, followed by addition of 10 mM sulfanilamide and 7 mM α-napthyl amine, and the absorbance was recorded at 530 nm and plotted using NO_2_^−^ as standard. For the determination of H_2_O_2_ accumulation, plant tissues were homogenized with 3 mL of 1% (*w*/*v*) tricarboxylic acid (TCA) solution, followed by centrifugation at 10,000× *g* for 15 min at 4 °C. An aliquot of the supernatant was incubated with 0.5 mM potassium iodide and 10 mM phosphate buffer (pH 7.0) for 30 min. The absorbance was read at 390 nm and plotted using H_2_O_2_ as standard. 

### 2.8. Assay of Antioxidative Enzymes and Their Polymorphisms

An assay of the antioxidative enzymes, APX (EC: 1.11.1.11) and GST (EC: 2.5.1.18), both in vivo and in vitro, was done according to Khatun et al. [39]. For APX activity, samples were homogenized in 50 mM phosphate buffer (pH 7.0) containing 1 mM EDTA, 1% (*w*/*v*) PVP, and 1 mM ascorbic acid following centrifugation at 15,000× *g*, 15 min, 4 °C. Thereafter, the enzyme activity was determined spectrophotometrically at 290 nm after the oxidation of ascorbate to dehydroascorbate. For GST activity, samples were homogenized in Tris-HCL buffer (pH 7.4) following centrifugation at 13,000× *g*, 15 min, 4 °C. An aliquot of the supernatant was reacted with 0.20 M potassium phosphate (pH 6.5), 0.1 mM DTT, 20 mM GSH, 0.10 M 1-chloro-2,4-dinitrobenzene (CDNB), and 0.01 M dimethyl sulfoxide (DMSO) following incubation for 5 min at 37 °C and the absorbance was read at 340 nm. For the polymorphic analysis of APX and GST through in-gel analysis, the extracted enzyme was run on a 10% non SDS-PAGE at 10 mV/lane in 50 mM Tris-HCl buffer (pH 6.8) and detection of isoforms was done in respective staining buffer. After that, photographs of the gel were taken in Gel Doc instrument with high resolution following densitometric analysis (Gel Analyzer software (Version-19.1)).

### 2.9. Statistical Analysis

All the data were initially subjected to student’s *t*-test by SPSS software (IBM, Armonk, New York, NY, USA). The principal component analysis (PCA) and correlation matrix were done using Origin2020b software (Origin Lab, Northampton, MA USA).

## 3. Result

### 3.1. Accumulation of Released NO (from SNP) and ABA Content

**Figure 1:**
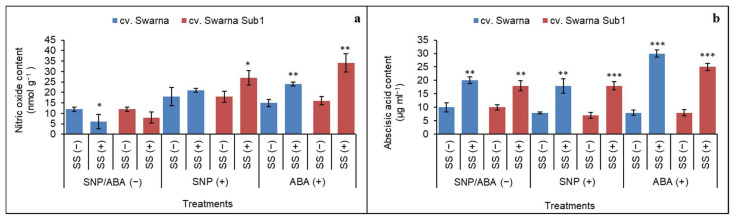
Changes in nitric oxide (**a**) and abscisic acid (**b**) contents in two rice genotypes (cv. Swarna and Swarna Sub1) under salinity stress [SS (+)] and non-salinity stress [SS (−)] for 72 h with [SNP (+) or ABA (+)] or without [SNP/ABA (−)] priming for 24 h. Data are presented in bars with mean ± SE (*n* = 3) from independent experimental sets and the significant values are represented as * (*p* ≤ 0.05), ** (*p* ≤ 0.01) and *** (*p* ≤ 0.001) by student’s *t*-test to denote the significant difference between SS (−) and SS (+) of SNP/ABA (−) as well as SNP (+) and ABA (+) of each rice genotype.

Salt stress can alter the biosynthesis of NO and ABA in a discriminatory manner after priming with [SNP/ABA (−)] (Figure 1a,b). Thus, irrespective of rice cultivar, the plants reduced accumulation of NO by 55 % and 45% for cv Swarna and Swarna Sub1 under salt stress [SS (+)] over non-salt stress [SS (-)] when no priming was done (Figure 1a). This trend was reverted when SNP (+) and ABA (+) were exogenously applied regardless of the rice cultivar. Thus, ABA (+) showed maximally retrieved NO accumulation by 2.1 fold in cv Swarna Sub1 than cv Swarna for 0.8 fold. In contrast, for ABA, the trend of accumulation was quite distinct with a rise irrespective of priming or non-priming by ABA/SNP. Thus, the maximum rise in ABA was observed by 4.2 and 3.5 fold in cv Swarna and cv Swarna Sub1 under ABA (+), which was the same as the non-priming set. SNP(+) had the same trend for ABA accumulation, where cv Swarna and Swarna Sub1 recorded 1.9 and 1.4 fold higher content respectively. Apparently, on the varietal basis, cv. Swarna Sub1 scored maximum values of NO and for ABA it was cv. Swarna Sub1, regardless of the type of priming agent.

### 3.2. Total PA Bioaccumulation as a Function of SNP and ABA Priming

**Figure 2:**
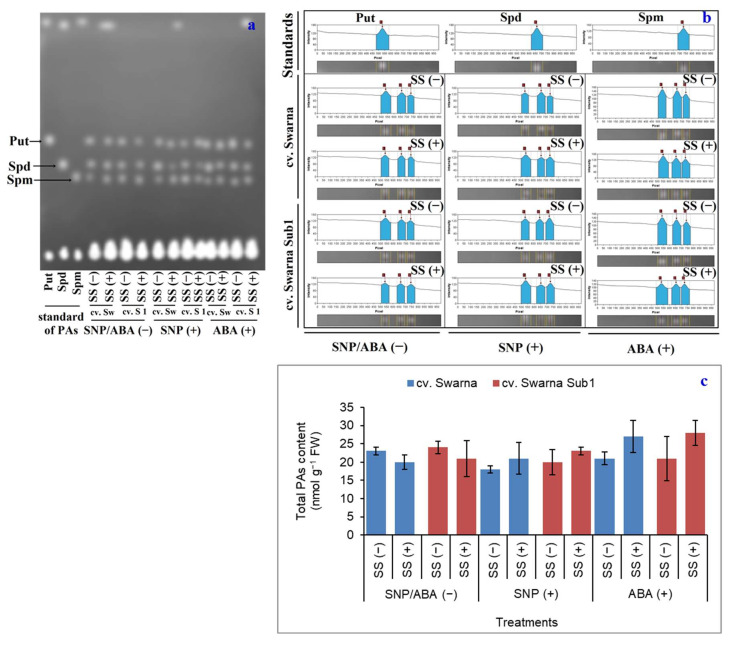
Separation and distribution of polyamines (Put- Putrescine, Spd- Spermidine, Spm- Spermine) from plant extract through thin-layer paper chromatography (**a**), its densitometric analysis (**b**) and quantification (**c**) in two rice genotypes (cv. Swarna and Swarna Sub1) under salinity stress [SS (+)] and non-salinity stress [SS (−)] for 72 h with [SNP (+) or ABA (+)] or without [SNP/ABA (−)] priming for 24 h. Data are presented in bars with mean ± SE (*n* = 3) from independent experimental sets and the significant values are represented as * (*p* ≤ 0.05), ** (*p* ≤ 0.01) and *** (*p* ≤ 0.001) by student’s *t*-test to denote the significant difference between SS (−) and SS (+) of SNP/ABA (−) as well as SNP (+) and ABA (+) of each rice genotype.

The total amount of PA was isolated from plant tissues of rice cultivars subjected to salinity as well as SNP and ABA priming, which were separated on TLC chromatogram and densitometric scanning and made to detect the relative abundance (Figure 2a,b). Under SS (+) with no exogenous priming, plants responded in a down-regulatory manner for total PA content irrespective of cultivar. SNP (+) and ABA (+) recovered an average of 16% and 31% PA content, respectively, under SS (+) against SS (−), irrespective of the rice cultivar (Figure 2c). Therefore, PAs are considered to have significant roles in salinity tolerance, however, they should not be considered as a contributing factor through NO and ABA moderation as observed in the present experiment.

### 3.3. Regulation of Water Status and Variation in Ionic Concentration under Salinity

**Figure 3:**
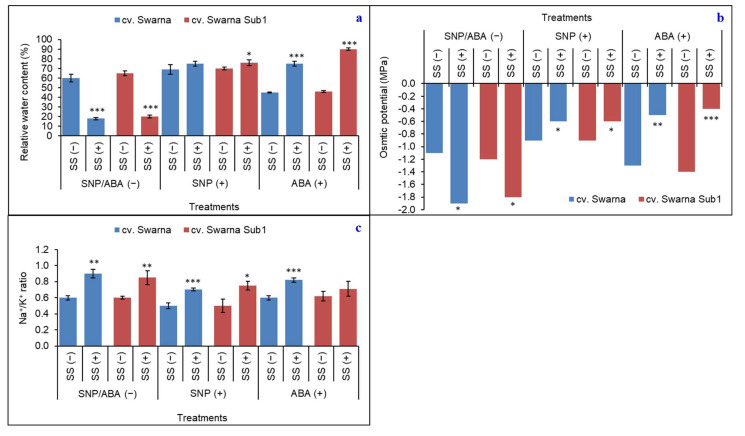
Changes in relative water content (**a**), osmotic potential (**b**) and Na^+^/K^+^ (**c**) in two rice genotypes (cv. Swarna and Swarna Sub1) under salinity stress [SS (+)] and non-salinity stress [SS (−)] for 72 h with [SNP (+) or ABA (+)] or without [SNP/ABA (−)] priming for 24 h. Data are presented in bars with mean ± SE (*n* = 3) from independent experimental sets and the significant values are represented as * (*p* ≤ 0.05), ** (*p* ≤ 0.01) and *** (*p* ≤ 0.001) by student’s *t*-test to denote the significant difference between SS (−) and SS (+) of SNP/ABA (−) as well as SNP (+) and ABA (+) of each rice genotype.

Under salinity with no priming, the RWC and OP of seedlings were reduced by 70% and 62%, respectively, irrespective of rice cultivar (Figure 3a,b). On the other hand, plants could retrieve RWC on average by 9% and 82%, respectively, with SNP (+) and ABA (+), irrespective of rice cultivar. The same trend was also noticed for OP, where the values were 33% and 105%, respectively. Thus, ABA (+) appeared promising in maintaining the water status in rice seedlings but SNP improved water status in all conditions regardless of stress, and therefore could also be another promising agent in osmotic conservation. It was observed that cv. Swarna Sub1 was better at responding to osmotic activities induced by salinity [SS (+)] since it maintained a higher value of RWC through both NO and ABA. The increase in Na^+^/K^+^ in non-primed plants was 46% under stress, whereas, it was 45% and 26% with SNP (+) and ABA (+), respectively, irrespective of cultivar (Figure 3c).

### 3.4. Variable Responses of ALA, GABA and GB

**Figure 4:**
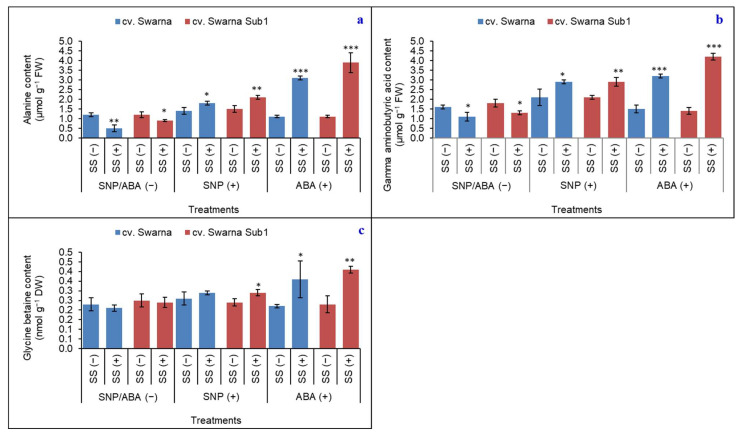
Changes in alanine (**a**), gamma aminobutyric acid (**b**) and glycine betaine (**c**) contents in two rice genotypes (cv. Swarna and Swarna Sub1) under salinity stress [SS (+)] and non-salinity stress [SS (−)] for 72 h with [SNP (+) or ABA (+)] or without [SNP/ABA (−)] priming for 24 h. Data are presented in bars with mean ± SE (*n* = 3) from independent experimental sets and the significant values are represented as * (*p* ≤ 0.05), ** (*p* ≤ 0.01) and *** (*p* ≤ 0.001) by student’s *t*-test to denote the significant difference between SS (−) and SS (+) of SNP/ABA (−), as well as SNP (+) and ABA (+) of each rice genotype.

The result showed that the concentration of compatible solutes (ALA, GABA, and GB) and their concentrations were regulated by salinity (Figure 4a–c). Plants failed to lose their osmotic turgidity as recorded by subdued concentration for ALA (58%), GABA (31%), and GB (9%) with SNP/ABA (−) under SS (+) for cv. Swarna. For cv. Swarna Sub1, the subdued concentrations were 25%, 28%, and 4%, respectively. On exposure to SNP and ABA, [SNP (+) and ABA (+)] plants retrieved those metabolites irrespective of rice cultivars; however, the maximum was seen in cv. Swarna Sub1. On a comparative basis, ALA and GABA contributed more (by 17% and 71%, and 38% and 156% increase) under SS (+) with SNP (+) and ABA (+), respectively, regardless of cultivar. GB was promising only with ABA (+), rather than SNP (+), for both cultivars.

### 3.5. NADP-ME, Sucrose, and Related Metabolizing Enzymes

**Figure 5:**
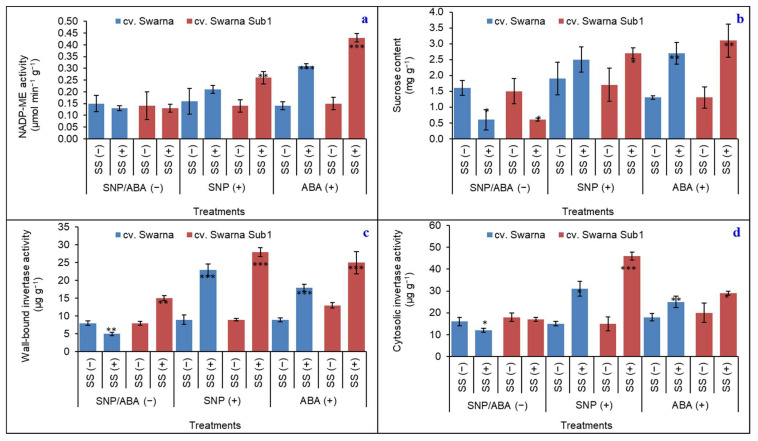
Changes in NADP-ME activity (**a**), sucrose content (**b**), wall-bound invertase activity (**c**) and cytosolic invertase activity (**d**) in two rice genotypes (cv. Swarna and Swarna Sub1) under salinity stress [SS (+)] and non-salinity stress [SS (−)] for 72 h with [SNP (+) or ABA (+)] or without [SNP/ABA (−)] priming for 24 h. Data are presented in bars with mean ± SE (*n* = 3) from independent experimental sets and the significant values are represented as * (*p* ≤ 0.05), ** (*p* ≤ 0.01) and *** (*p* ≤ 0.001) by student’s *t*-test to denote the significant difference between SS (−) and SS (+) of SNP/ABA (−) as well as SNP (+) and ABA (+) of each rice genotype.

NADP-ME, which represents an alternative CO_2_ source through decarboxylation reaction, was more pronounced in cv. Swarna Sub1 than cv. Swarna both in primed and non-primed plants. On account of SNP (+), the activity promisingly increased by 86% only in cv. Swarna Sub1 under SS (+) against SS (−) (Figure 5a). However, with ABA (+), the activity promisingly increased for both cv. Swarna and Swarna Sub1 and the values were 121% and 186%, respectively. This might suggest that both chemical primings had their respective effects on cv. Swarna Sub1 by gaining carboxylation through the decarboxylation pathway, which may compensate for the loss of photosynthetic activity under saline stress. On account of photosynthetic carbon acquisition, sucrose and its major metabolizing enzymes including invertase (wall-bound and cytosolic) were considered from rice seedlings under SS (+) with SNP (+) and ABA (+) thereon. A steady decline of sucrose concentration of the tissues of plants by 62% and 60% under SS (+) with SNP/ABA (−) in cv. Swarna and Swarna Sub1, respectively, was recorded (Figure 5b). However, the content promisingly increased in cv. Swarna Sub1 with both chemical elicitors. Moreover, major sucrose metabolizing enzymes isolated and purified from the cell wall and cytosol also followed an almost down-regulatory trend for both cultivars in non-primed plants (Figure 5c,d). Meanwhile, with SNP (+) and ABA (+), the activities were retrieved by 170% and 69%, respectively, on average of enzyme sources, irrespective of cultivar. 

### 3.6. Variations in Polymorphic Expression of NADP-ME and Related Metabolizing Enzymes

**Figure 6:**
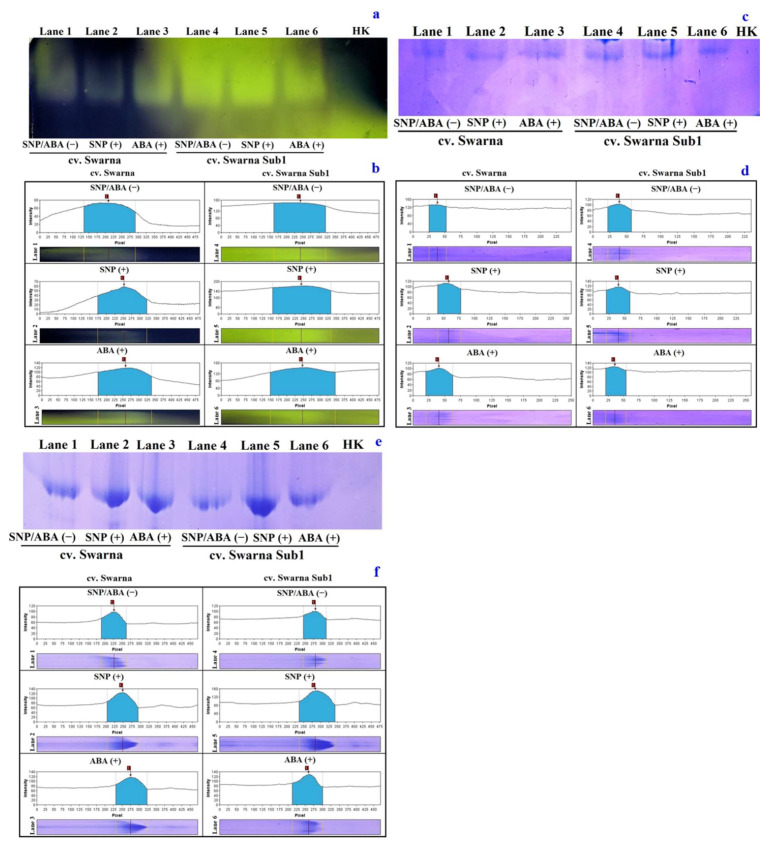
Polymorphic expression profile and densitometric analysis of NADP-ME activity ((**a**,**b**), respectively), wall-bound invertase activity ((**c**,**d**), respectively), cytosolic invertase activity ((**e**,**f**), respectively) in two rice genotypes (cv. Swarna and Swarna Sub1) under salinity stress [SS (+)] and non-salinity stress [SS (−)] for 72 h with [SNP (+) or ABA (+)] or without [SNP/ABA (−)] priming for 24 h. Loading plot: HK- heat killed.

On isozymic polymorphic expression for NADP-ME, a single but distinct band featured with more expression under SS (+) with chemical priming for both the cultivars (Figure 6a). The densitometric images also showed the variation of polypeptide concentrations as resolved through native gel, which clearly suggested the involvement of chemical signaling (SNP and ABA) for decarboxylation (Figure 6b). However, maximum intensities of polypeptide through a single band were scored with ABA (+) rather than SNP (+). The polymorphic expression profile and its densitometric analysis of invertase as a protein isolated from apoplastic (cell wall) and symplastic (cytosolic) fraction revealed hardly any variation (Figure 6c–f). However, the intensity of the bands from the polymorphic protein was denser in the case of ABA (+) than SNP (+) in both cultivars. Furthermore, the impact of salinity stress ensured a better tolerance for respective cultivars from both differentially cited invertase enzymes in the plants.

### 3.7. Variation in Path of ROS Generation and Its Polymorphism

**Figure 7:**
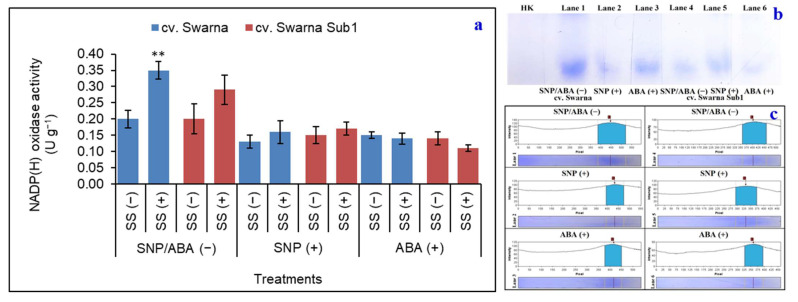
Changes in NADP(H) oxidase activity (**a**) and its polymorphic expression profile and densitometric analysis ((**b**) and (**c**), respectively) in two rice genotypes (cv. Swarna and Swarna Sub1) under salinity stress [SS (+)] and non-salinity stress [SS (−)] for 72 h with [SNP (+) or ABA (+)] or without [SNP/ABA (−)] priming for 24 h. Data are presented in bars with mean ± SE (*n* = 3) from independent experimental sets and the significant values are represented as * (*p* ≤ 0.05), ** (*p* ≤ 0.01) and *** (*p* ≤ 0.001) by student’s *t*-test to denote the significant difference between SS (−) and SS (+) of SNP/ABA (−) as well as SNP (+) and ABA (+) of each rice genotype. Loading plot: HK-heat killed.

For estimation of oxidative status, rice seedlings from salinity treatment were compared with variants of ROS like O_2_**^•^**^−^ and H_2_O_2_ and their biosynthetic pathways. Thus, NOX activity showed a steady increase under SS (+) proportionally by 75% and 45% in cv. Swarna and cv. Swarna Sub1, respectively, in non-primed plants (Figure 7a). This was also supported by the band intensities and their densitometric analysis (Figure 7b,c). Therefore, the path of ROS generation may be the key factor for reactions of rice seedlings to the salinity when compared to control.

### 3.8. Variation in ROS Content and Its Histochemistry

**Figure 8:**
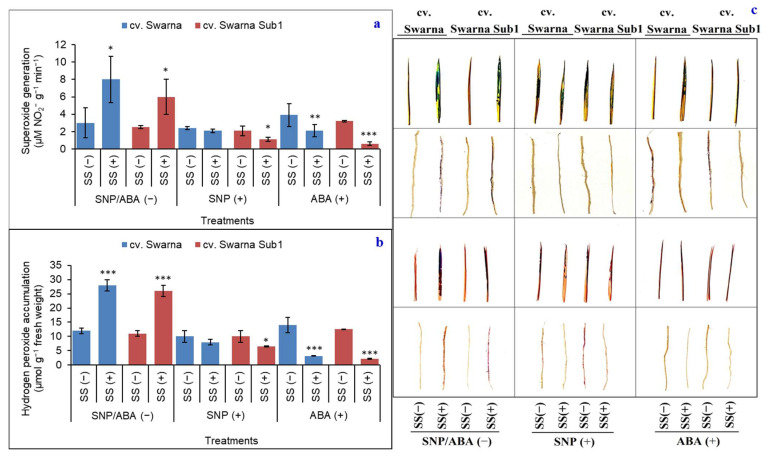
Changes in superoxide generation (**a**), hydrogen peroxide accumulation (**b**) and their histochemical detection in leaves and roots (**c**) of two rice genotypes (cv. Swarna and Swarna Sub1) under salinity stress [SS (+)] and non-salinity stress [SS (−)] for 72 h with [SNP (+) or ABA (+)] or without [SNP/ABA (−)] priming for 24 h. Data are presented in bars with mean ± SE (*n* = 3) from independent experimental sets and the significant values are represented as * (*p* ≤ 0.05), ** (*p* ≤ 0.01) and *** (*p* ≤ 0.001) by student’s *t*-test to denote the significant difference between SS (−) and SS (+) of SNP/ABA (−) as well as SNP (+) and ABA (+) of each rice genotype.

The products of NOX such as O_2_**^•−^** showed a similar trend, increased by 167% and 140% for those cultivars, respectively (Figure 8a). H_2_O_2_, another ROS, showed the same trend under SS (+) in non-primed plants (Figure 8b). On average, the subdued values of both O_2_**^•^**^−^ and H_2_O_2_ were 30% and 28% with SNP (+), and 64% and 80% with ABA (+), irrespective of cultivar. Moreover, the control of oxidative stress was more pronounced for cv. Swarna Sub1 for both the ROS generating pathway and its variants under SS (+) than cv. Swarna. Distribution of ROS when observed histochemically in leaves and roots showed more accumulation under SS (+) with SNP/ABA (−) (Figure 8c). The pattern of ROS accumulation was mainly at the leaf margin, extending towards the center for O_2_**^•^**^−^ and H_2_O_2_, which were diffuse and scant in the case of roots under SS (+). However, cv. Swarna Sub1 recorded a more reduced accumulation of O_2_**^•^**^−^ and H_2_O_2_ with both SNP (+) and ABA (+), which was also comparable for cv. Swarna but at a smaller magnitude.

### 3.9. Activities of APX and GST 

**Figure 9:**
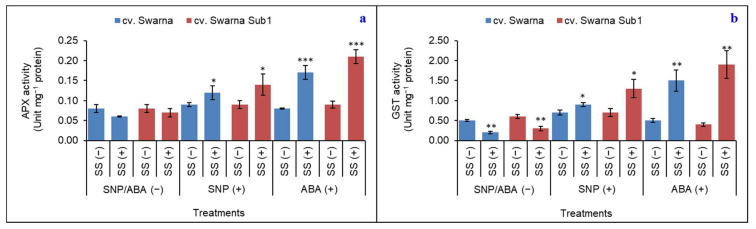
Changes in ascorbate peroxidase (**a**) and glutathione *S*-transferase (**b**) activity in two rice genotypes (cv. Swarna and Swarna Sub1) under salinity stress [SS (+)] and non-salinity stress [SS (−)] for 72 h with [SNP (+) or ABA (+)] or without [SNP/ABA (−)] priming for 24 h. Data are presented in bars with mean ± SE (*n* = 3) from independent experimental sets and the significant values are represented as * (*p* ≤ 0.05), ** (*p* ≤ 0.01) and *** (*p* ≤ 0.001) by student’s *t*-test to denote the significant difference between SS (−) and SS (+) of SNP/ABA (−) as well as SNP (+) and ABA (+) of each rice genotype.

On average, APX activity showed a declining trend (19% decrease) regardless of rice cultivar. SNP (+) and ABA (+) rice seedlings showed increased activity by 45 and 123%, respectively, irrespective of the cultivar (Figure 9a). Plants sequestered metallic ion in the form of Na^+^ during the experiment, which was sequestered into the vacuole by GST activity. In this way, the rice cultivars increased GST activity irrespective of cultivar by 55% under SS (+) with SNP/ABA (−) (Figure 9b). SNP (+) and ABA (+) caused up-regulation by 57% and 288% respectively, irrespective of cultivar. On a comparative basis, cv. Swarna Sub1 experienced a better response to both SNP (+) and ABA (+) in sequestering the Na^+^ compared to the other one.

### 3.10. Variation in Polymorphic Expression Profile of APX and GST

**Figure 10:**
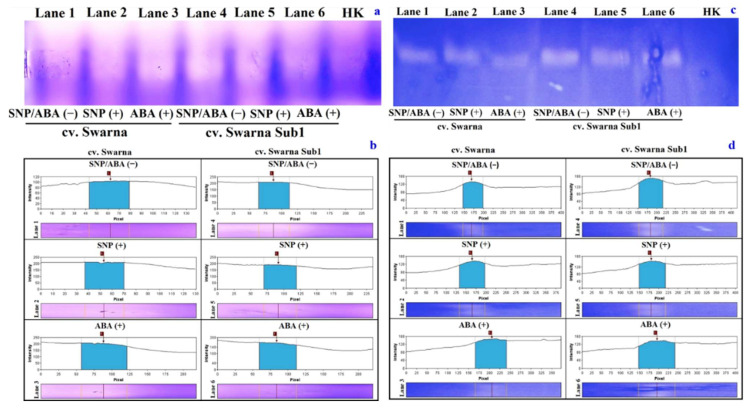
Changes in polymorphisms of ascorbate peroxidase and glutathione S-transferase ((**a**,**c**), respectively) and their densitometric analysis ((**b**,**d**), respectively) in two rice genotypes (cv. Swarna and Swarna Sub1) under salinity stress [SS (+)] and non-salinity stress [SS (−)] for 72 h with [SNP (+) or ABA (+)] or without [SNP/ABA (−)] priming for 24 h. Loading plot: HK- heat killed.

The same trend as the in vitro analysis was followed in the polymorphic expression profile of APX activity and its densitometric analysis (Figure 10a,b). However, the expression profile in polymorphic forms and densitometric scanning of GST recorded no variation between the cultivars but intensities of bands were distinct under SS (+) in non-primed plants (Figure 10c,d). A better concentration of polypeptide was observed under SS (+) with both SNP (+) and ABA (+).

### 3.11. Statistical Interpretation through PCA and Correlation Study

**Figure 11:**
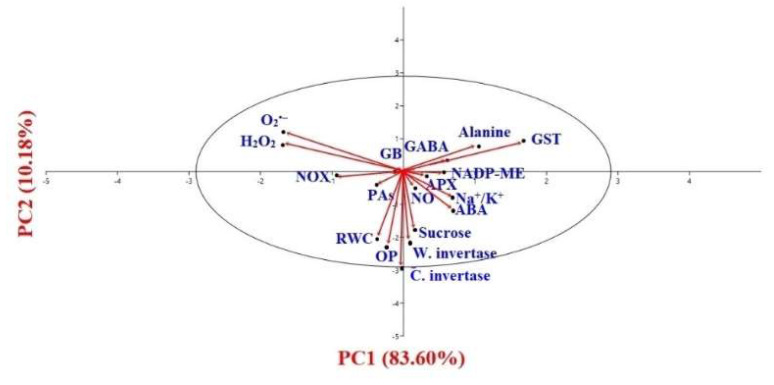
Two-dimensional scatter diagram of principal component analysis based on the first and second principal component of different attributes performed in the experiment. Loading plot: NO—nitric oxide content, ABA—abscisic acid content, Pas—total polyamines content, RWC—relative water content, OP—osmotic potential, Na^+^/K^+^—ratio of Na^+^ and K^+^, Alanine—alanine content, GABA—gamma aminobutyric acid content, GB—glycine betaine content, NADP-ME—NADP-malic enzyme activity, Sucrose—sucrose content, W. invertase–wall-bound invertase activity, C. invertase—cytosolic invertase activity, NOX—NADP(H) oxidase activity, O_2_**^•^**^−^—superoxide generation, H_2_O_2_—hydrogen peroxide accumulation, APX—ascorbate peroxidase activity, GST—glutathione *S*-transferase activity.

The biplot of PCA was represented by its two main components PC1 and PC2 (Figure 11). PC1 and PC2 scored 84% and 10% of the total variability, respectively. Thus, the total cumulative variance of these components accounted for about 94%. Under the salinity stress condition and irrespective of chemical elicitors, PC1 demonstrated a positive correlation with NO, ABA, ALA, GABA, NADP-ME, APX, GST, Na^+^/K^+^, sucrose, wall-bound, and cytosolic invertase activity. On the other hand, another component, PC2, represented the positive correlation only with O_2_**^•−^**, H_2_O_2,_ and GB.

**Figure 12:**
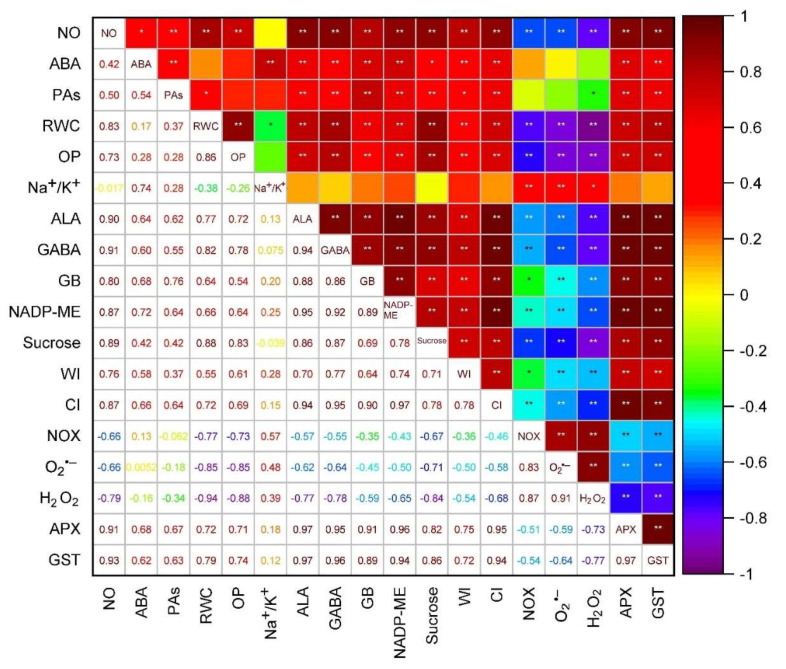
The Pearson’s correlation matrix among the parameters performed in the experiment. Loading plot: NO—nitric oxide content, ABA—abscisic acid content, PAs—total polyamines content, RWC—relative water content, OP—osmotic potential, Na^+^/K^+^—ratio of Na^+^and K^+^, Alanine—alanine content, GABA—gamma aminobutyric acid content, GB—glycine betaine content, NADP-ME—NADP-malic enzyme activity, Sucrose—sucrose content, WI−wall-bound invertase activity, CI—cytosolic invertase activity, NOX—NADP(H) oxidase activity, O_2_**^•^**^−^—superoxide generation, H_2_O_2_—hydrogen peroxide accumulation, APX—ascorbate peroxidase activity, GST—glutathione *S*-transferase activity, * *p* ≤ 0.05, ** *p* ≤ 0.01.

From the correlation analysis, it was revealed that content of NO and ABA were correlated positively (r = +0.42) (Figure 12). Moreover, total PA content also showed a positive correlation with both the NO (r = +0.50) and ABA (r = +0.54) content. Regarding the water status attributes like RWC and OP, a positive correlation (r = +0.86) between them was found. ALA, GABA and GB also showed a positive correlation among them. The same trend was followed for wall bound and cellular invertase (r = +0.78) and O_2_**^•^**^−^ and H_2_O_2_ (r = +0.91).

## 4. Discussion

For a long time, an economic, biocompatible, and efficient control measure against salinity toxicity has been in practice in rice culture. *sub1A*, the prime factor exercised in rice cultivar in the present experiment, can augment ABA sensitivity when overexpressed, as reported earlier [40]. This also paradoxically induces dehydration upon the post-submergence period with leaf desiccation through significant ABA biosynthesis. Exogenously applied ABA has already proved to be variably useful for practical use in the recovery of salinity and water stresses with rice cultivars. The usefulness of SNP and ABA recorded a distinct variation through the functioning of *sub1A* QTL; however, differentially in cv. Swarna and Swarna Sub1 (Supplementary Figure S1). *sub1A* response elements in selective waterlogged rice genotypes have been shown to share compatibility with ABA responsiveness. In fact, this is more prudent when rice genotypes are faced with excess salt accumulation-related toxicity. Under abiotic stress, NO benefit rice with regards to *sub1A* sensitivity, albeit differentially. However, it has not been dispensed, particularly, under salt-induced dehydration. *sub1A* QTL basically coordinates ET metabolism, particularly, anoxic sensing of O_2_ sharing NO and ABA signaling as usual under water deficit. Therefore, RNS and ABA may be prudent for the assessment of cellular and physiological responses as described in the present experiment.

### 4.1. ABA and SNP Priming Influence Salinity Tolerance Variably

The effects of different residues on modification of plant responses are dependent on the nature of the chemical compound, reactivity, compatibility to cellular processes, distribution, etc. Those organic residues, if adjusted to the cellular environment after the application, become biocompatible and could modify the perception of stress signaling from the environment or/and alter the stress responses in plant cells. Likewise, PAs and ABA are ubiquitously present in plants and widely offered for stress tolerance in different physiological perspectives [41]. In contrast, NO is less abundant in the cellular environment and the imbalances of nitrate metabolism are mainly the primary source for signaling. However, PAs and ABA are mostly biocompatible in regards to their salinity tolerance and have a common function with osmotic compatible solutes to maintain cellular turgidity. However, ABA is more related to the influence on stomatal physiology and has an important role in K^+^ uptake and development of other compatible solutes (proline, sucrose). PAs however are more related to mechanical shielding of the membrane and other cellular organelles due to electrostatic binding of their positively charged NH_2_ groups to other negatively charged residues of cells. Therefore, plants’ tolerance through PAs, as expected from other cases, was also satisfied irrespective of rice cultivar in this experiment of over-production of content under salinity. NO and ABA may directly or indirectly influence the biosynthesis of PAs either by regulating any of the rate-limiting genes (S-adenosine methionine decarboxylase, etc.) or limitation of PA catabolism [42]. In rice, particularly under salinity, ABA-mediated tolerance has also been well documented in the overexpression of the S-adenosylmethionine decarboxylase (SAM-DC) gene, but with variability in tolerant and susceptible cultivars. PA catabolism, with its oxidation into H_2_O_2_, is another pathway of tolerance, and, in particular, downstream signaling for antioxidation is important [43]. ABA, despite its anti-dehydration nature, still strongly regulates PAs regardless of water and salinity stress. Few genes are synchronized in their expression for the regulation of PAs and their turnover. For PA degradation by amino-oxidase, well-functioning ABA and NO control has been reported in plants. Therefore, the accumulation and depletion in a significantly higher mode in cv. Swarna Sub1 might be more due to catabolism of PAs compared to other cultivars under salinity.

### 4.2. NO and ABA May Maintain the Osmotic Balance in Plants under Salinity

As compared to control plants, cv. Swarna *Sub1* was more tolerant to salinity stress than others in this experiment. Salinity is minimized by retaliation water relation parameters like water potential, root hydraulic conductivity, relative water content, and membrane permeability [44]. cv. Swarna Sub1 has better impacts with NO and ABA through RWC and tissue osmotic potential. In rice, *sub1A* QTL, by adopting an ET-mediated quiescence strategy, maximizes the RWC to ensure adequate water absorption following tissue hydration [45]. In rice cultivars possessing the *sub1A* trait, sensing hypoxia is also realized under the condition of soil-O_2_-tension in salinity and similar recovery through a hydro active mechanism through ABA signaling is operative. Overproduction of ABA under salinity in the root and distribution through water conduits causes depolarization of the membrane, which is responsible for the opening of ion channels [46]. The latter may compensate for the influx of K^+^, which becomes more deficient under salinity stress and thereby, stabilizes the water relation [47]. In contrast, NO is more affiliated with rescuing the membrane integrity by minimizing the peroxidation reaction as well as inducing antioxidative activities under salinity stress. The impact of NO on stomatal regulation is well documented with the alteration of ROS metabolism on water stress with reference to ion efflux [48]. However, at the root level, the sensitivity of NO under NaCl stress shares both ABA-dependent and independent routes; however, a differentially expressed cascade of genes for tolerance has been proposed in rice roots [49]. The results were verified from a strong correlation with NO-primed seedlings irrespective of cv. Swarna and cv. Swarna Sub1 in the recovery of water status compared to non-primed stressed plants. This finding strongly suggests that exogenous application of NO ameliorates NaCl sensitivity through retention of tissue hydration, bearing conformity with wheat [50], cucumber [51], and Chinese cabbage [52] under stress.

### 4.3. SNP and ABA Regulate Compatible Solutes, Metallic Ion, Photosynthetic Decarboxylation, and Sucrose Metabolism under Salinity 

Under salinity, compatible solutes are typically required for maintaining the osmotic balance due to Na^+^ accumulated osmotic perturbances. In the present experiment, cv. Swarna Sub1 experienced a steadier increase in ALA, GABA, and GB compared to cv. Swarna. Compatible solutes with differential accumulation in tolerant and sensitive genotypes ensure the osmotic turgidity, ion homeostasis, membrane ion integrity, and antioxidation [53]. NO and ABA might regain depleted hydration under salinity with overproduction of ALA, GABA, and GB variably in cv. Swarna and Swarna Sub1. Under inundation, *sub1A* QTL also showed responsiveness in overexpressed synthesis of osmolytes like proline in support to plants’ growth. The improved synthesis of protein, functioning of enzymes, fortification of membrane native structure, and antioxidation support growth [54]. Therefore, identification of possible paths in *sub1A* QTL bearing rice genotypes would be effective against salinity stress through receptiveness to NO. A similar increase in GB and GABA strengthened the osmotic performances under salinity, withholding K^+^ balance against Na^+^ with exogenous administration of NO and ABA under salinity [55], heat [56], and chilling [57] stress. In rice and other crops, both NO and ABA have successfully been shown to retrieve photosynthetic assimilation. This is based on modulation of carbon reduction metabolism as well as efficiency of photosynthetic light through fluorescence characteristics. The latter maintains integrity in the chloroplast membrane and thereby its energy transduction through photosystems. Decarboxylation with organic acids by the enzymatic system is the most important, where NADP-ME has importantly been studied under salinity stress [58]. Likewise, the activity of NADP-ME in the present experiment is expected to have some relief on rice cultivars under salinity with induction of NO and ABA priming. Under salinity-induced water stress, decarboxylation on malic acid by NADP-ME releases CO_2_ with H^+^ establishing in the stroma with higher pH. This favors more transportation of Mg^2+^ from the cytosol, favoring the activity of Calvin cycle enzymes, but this is exclusively for the C_4_ system. Non-photosynthetic NADP-ME in rice also contributes a reducing equivalent (NADPH + H^+^). This is mostly required for lipids, secondary metabolites, and oxidative pentose phosphate pathways biosynthetic pools [59]. ABA induces the malate/OH transporter and thereby increases the accessibility for malate accumulation. In rice under salinity, malate metabolism undergoes anaplerotic reactions where released CO_2_ might be involved in the biosynthesis of compatible solutes and other intermediates. Interestingly, both the rice cultivars herein favored sustained NADP-ME activity more with ABA than NO. NO, with its ample possibility, could be involved in the reversal of oxidative damage, sustaining enzyme integrity. Under salinity, plants are otherwise more sensitive to ET- and glucose-induced suppressed photosynthesis. Excess glucose accumulation would be key to the feedback inhibition for the carboxylase reaction, where NO acts as reliever [60]. On that basis, *sub1A* QTL in Swarna Sub1 with ET sensing activity might be better in the sustenance of NADP-ME by NO priming than cv. Swarna under salinity.

On the other side, NO and ABA, as identified key modulators, dependently/independently influence the starch–sucrose interrelationship under salinity stress in plants [61]. Sucrose metabolism happens to be an adaptive measure through carbon allocation against salinity and alkalinity with a continued flux of sucrose hydrolysis into reducing sugars. The activity of invertase over sucrose into simpler sugars is utilized mostly for glycolytic paths in support of growth and maintenance of respiration. The latter is effective for tissue viability, particularly when photosynthetic carbon fixation is restricted [62]. ABA exerts its effect on the upregulation of invertase activity in active photosynthesizing tissues when stomata are closed under salinity. Under this condition, the reducing sugars, under the activity of cell wall-bound invertase, replenish glycolytic flux and its sustenance [63]. On the other hand, translocation of sucrose for maintenance of the source sink continuum is important under salinity as a selective feature for sustained photosynthetic activities in rice [64]. cv. Swarna Sub1 was observed to perform sucrose metabolism better through more leniency to NO and ABA than the other cultivar. Out of two metallic ions (Na^+^ and K^+^), plants are more sensitive to loss of K^+^ than the other one. Under salinity stress, the primary feature recorded in plants is dehydration through loss of turgidity of tissues. Undoubtedly, K^+^ accumulation by roots and its distribution through conductive tissues to other plant parts is influenced by membrane functions. K^+^ uptake under salinity in plant roots is mostly mediated by the high K^+^ transporter (HKT1, HKT2) and secondarily by ion pumps (H^+^/ATPase). In an earlier experiment, we found increased activity of H^+^/ATPase following metal accumulation over the membrane, but this was under metalloid stress. Thus, the overexpression of H^+^/ATPase activity and a higher ratio of K^+^ intrusion to Na^+^ by both NO and ABA is interesting in the present experiment. This is because rice seedlings were shown to recover from salinity-induced detrimental effects (mostly retrieving the osmotic turgidity) by priming of SNP and ABA.

### 4.4. NO and ABA Independently Moderate the Oxidative Stress under Salinity

NO sensitivity significantly varies with crop species, duration of stress revelation, presence of other elicitors, tissue specificity, etc. In rice, root sensitizes more than shoot with NO sensitization and accumulation through nitrite reductase activity [65]. Chemical conversion of NO to other ROS-like peroxides is featured in rice roots under NH_4_^+^/NO_3_^−^ supplementation. Therefore, a simultaneous increase in the cellular concentration of NO and ROS would indicate dependency of oxidative stress through common possible pathways with NOX, a xanthin-like oxidase [66]. This is well depicted in the present experiment with a significant up-regulation of wall-bound NOX activity under salinity. In rice particularly, anaerobic or O_2_ deficiency probes a redox signaling through both NO and ROS through induction of VII ET responsive factor (ERF). An ERF has been shown to be compatible with water stress tolerance through the quiescence strategy by ethylene metabolism [67]. Moreover, NO signaling is also reflected in rice due to moderation of ethylene responses. Therefore, a prediction for the occupation of *sub1A* QTL, an ERF in favor of NO sensitivity in plants for altering the responses is expected to be prudent, and, under salinity where roots also sense hypoxia due to depleted soil, O_2_ tension may equally promote ROS generation through usual apoplastic/symplastic pathways. Therefore, cv. Swarna Sub1 with its over-expressed NOX activity may facilitate ROS generation for its oxidative degeneration of cellular residues. Support of NO in reducing the derivatives of ROS directly or/and inducing antioxidant generation for quenching of ROS energy are equally important. The distribution of tissue-specific ROS accumulation irrespective of cultivar but with differential tolerance could imply NO sensitivity of those cultivars in a differential manner. On a comparative basis, the ABA could exceed responses for NOX activity more than NO and thereby raise the chances for more oxidative stress but it is rescued by existing antioxidation paths [68]. An equal possibility for both NO and ABA-induced antioxidation, particularly at the level of leaves and roots for O_2_**^•^**^−^ and H_2_O_2_, was revealed by histochemical detection but without significant variation between cultivars. Meanwhile, H_2_O_2_ rather than O_2_**^•^**^−^ has a more beneficial role in rice plants under salinity, which might possibly establish a pathway for better tolerance in cv. Swarna *Sub1*.

### 4.5. Enzymatic Alleviation of Salinity: Antioxidation and Ion Sequestration

For metal exclusion, enzyme-mediated detoxification by a few special families of multi-functional proteins is evident for salinity detoxification. Glutathione S-transferases (GST) are exploited through the developmental process of plants when exposed to diverse toxic residues like metals, metalloids, and xenobiotics. GST, which is widely distributed in tissues, undergoes GSH-mediated electrophilic reactions with compounds and is converted into a more solubilized form [69]. Under salinity, different groups of GST render the conjugation of Na^+^ and allied cations and transfer them into extracellular or vacuolar spaces. GSTs have also been reported to be induced under abiotic stresses, even under the influences of allelopathic compounds [70]. In most cases, the Na^+^ and heavy metal-induced lipid peroxides, hydroperoxide and epoxide, are detoxicated along with auxin homeostasis cysteine turn-over, and senescence regulation [71]. Thus, in the present experiment, cv. Swarna Sub1 showed much up-regulated activities of GST and undoubtedly scored better indices for Na^+^ detoxification even with NO and ABA. ABA has also been reported in plants to co-induce GST along with osmotic homeostasis in serving dual tolerance against salinity-induced damages. In contrast, NO has not been shown to induce GST activity in earlier studies but a few cases reported transformation of reduced sulfur into simpler non-thiol residues. However, rice roots exposed to NO also alter the membrane permeability to sulfur uptake and downstream metabolic reduction into residues like cysteine [72]. In the course of enzymatic antioxidation, a class of peroxidases is attributed by APX with ascorbic acid as the electron donor to lysis of H_2_O_2_. Ascorbate-mediated peroxidase activity against ROS happens to be a key path in different subcellular compartments [73]. A significant variation also exists in plant species in APX activity and its trend through different stressors according to genotypic potential. The expression of APX encoding genes is differentially modulated by several abiotic stresses, mostly depending on duration, intensity, elicitation/induction factors, plant ages, etc. In general, plants register an upregulation in activity as a major H_2_O_2_ detoxifying protein ascorbate-glutathione cycle [74]. Evidence is more pronounced where both NO- and ABA-mediated induction of APX is more common in different crop species [75]. APX is more active mostly in chloroplasts where immediate scavenging of H_2_O_2_ becomes indispensable in sustaining the normal photosynthetic activity. Therefore, photosynthesis with suppressed status under salinity may identify an electron donor for peroxidase reaction as ascorbate, and thereby, it appears as a major antioxidant. In the present experiment, the modification of APX activities and its major cytosolic isoforms might symbolize an overexpressed antioxidation under both NO and ABA. However, ABA was more aggressive in governing APX regulation than NO irrespective of rice cultivar in this experiment.

## 5. Conclusions

**Figure 13:**
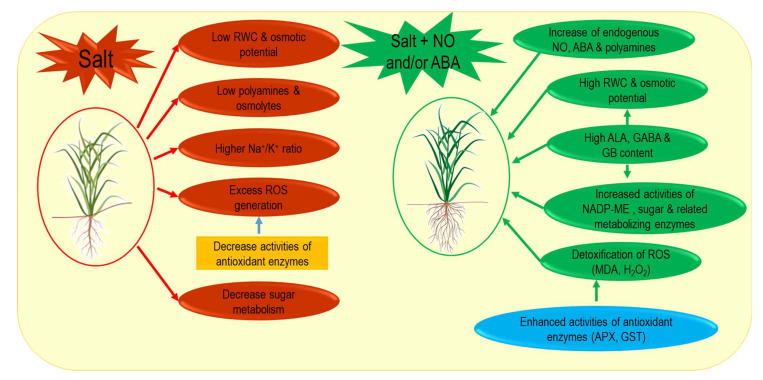
Summary of the protective role of NO and ABA in conferring salt tolerance in rice based on the present experimental results. Swarna was much more affected by salt compared to Swarna Sub1, while the protective effects of NO and/or ABA were more prominent in the cultivar Swarna Sub1.

From the present experiment, it is clearly evident that rice cultivars had equal chances to react in a differential manner to SNP and ABA interference. The physiological and cellular responses were more pronounced in regards to variability irrespective of which rice cultivar was exposed to salinity. The upregulated manner of sugar and sucrose hydrolysis supported plant tolerance initially under salinity. NO and ABA had distinct effects on these, suggesting a possible path of plants’ sensitivity to exogenous elicitation. Thus, the performance of ABA in osmotic maintenance might be complementary to NO in the same manner. This probably conceives the idea of an ABA independent/dependent NO route to secure the respective responses under salinity (Figure 13). Plants showed significant moderation of polyamines under both NO and ABA supplementation. Polyamines had reduced activity of peroxidation reactions on the membrane by regulation through O_2_**^•^**^−^ and H_2_O_2_. NO and ABA had their effects on apoplastic sugar metabolizing enzymes to secure stable photosynthetic flux. However, at the gene level, regulation of ABA induced distinct polymorphisms in enzymatic polypeptides in a better manner than NO. Therefore, polymorphism induction by both NO and ABA might be promising in the biomarking concept under salinity, but varied through cultivars. Additionally, decarboxylation reactions might support CO_2_ enrichment for plants through NADP-ME activity in acquisition of carbon for stress tolerance. This would be a basis for the formulation of NO/ABA-mediated salinity tolerance responses in rice where both oxidative stress and sugar metabolism are the prominent keys. From the focus of cultivar-specific responses, *sub1A* QTL seems to be the usual operative in ET modulation with ABA influencing different characters. In contrast, the non *sub1A* possessing rice cultivar responded less to ABA under the identical saline conditions. NO would otherwise be more differentiating in a manner contiguous with ABA, which may be suggestive that a complementary action of salinity tolerance was aided by ABA. Therefore, NO and ABA would be realistic in priming applications to secure salinity tolerance in rice culture. To the best of our knowledge, this study might realize the affectivity of NO and ABA at the metabolomics level, which in turn enriches the existing understanding of the cellular mechanism of salinity tolerance in rice.

## Data Availability

All data are available in this article.

## Supplementary Materials

The following are available online at https://www.mdpi.com/article/10.3390/plants11081084/s1, Figure S1: Growth of seedling in two rice genotypes Rice (cv Swarna and cv. Swarna Sub1) under salinity stress.
