# Supplementary material for: Differential Impact of Nitric Oxide and Abscisic Acid on the Cellular and Physiological Functioning of sub1A QTL Bearing Rice Genotype under Salt Stress
Source: Plants (Basel). 2022 Apr 15;11(8):1084. doi: 10.3390/plants11081084 (PMC9029218; doi:10.3390/plants11081084)
Supplement: Supplementary file 1 [file plants-11-01084-s001.zip › plants-1626863-supplementary.pdf]

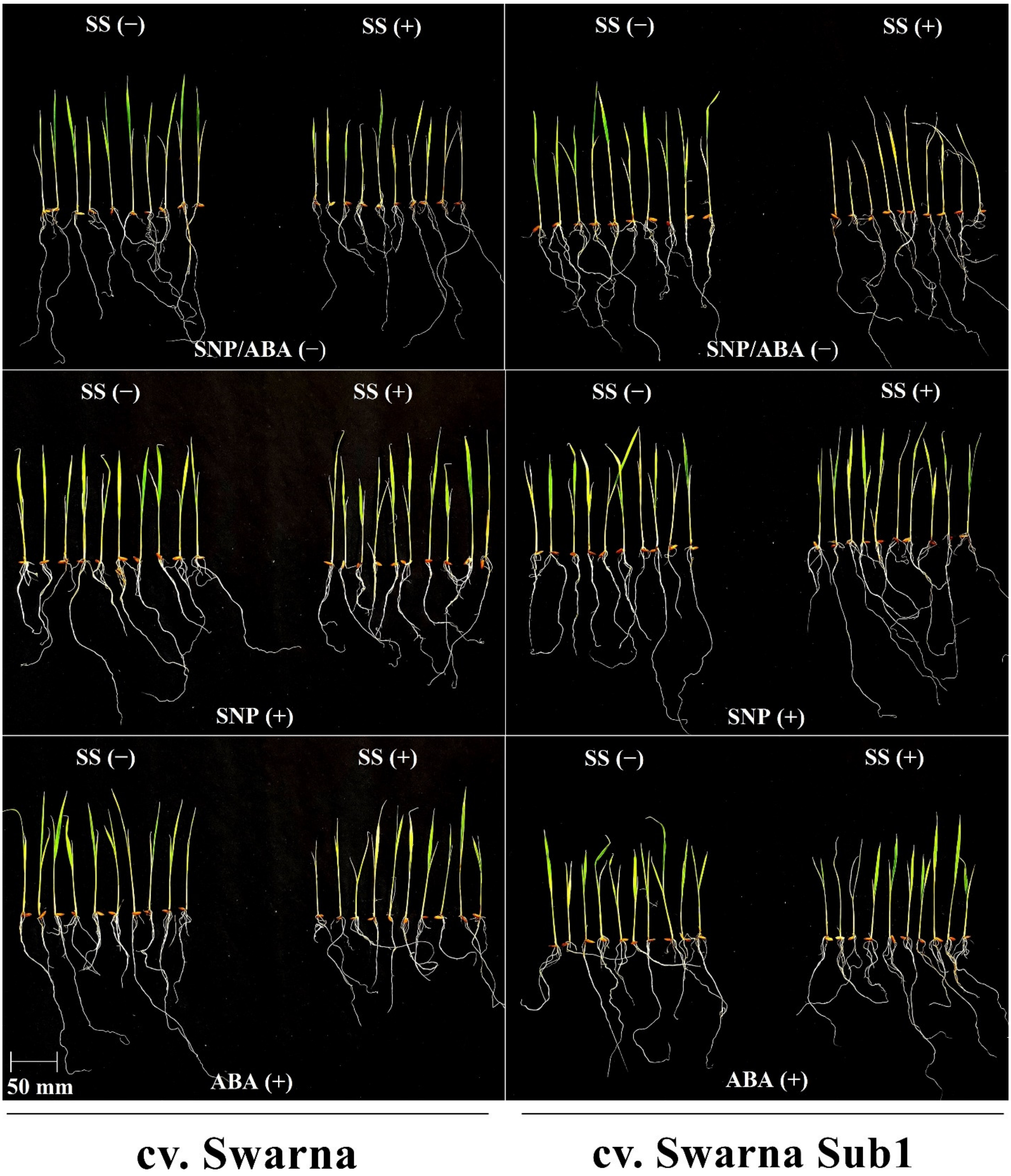

Figure S1. Growth of seedling in two rice genotypes Rice (cv Swarna and cv. Swarna Sub1) under salinity stress [SS (+)] and non-salinity stress [SS (-)] for 72h with [SNP (+) or ABA(+)] or without [SNP/ABA(-) priming for 24h.
